# Cost-effectiveness of a self-management maintenance programme following pulmonary rehabilitation: a UK randomised controlled trial for patients with chronic obstructive pulmonary disease

**DOI:** 10.1136/bmjresp-2025-003406

**Published:** 2025-12-04

**Authors:** Amir J Khan, Anil Gumber, Matthew Richardson, Claire M Marie Nolan, William D-C Man, Sally Singh, Linzy Houchen-Wolloff, Ala Szczepura

**Affiliations:** 1 Department of Economics, Institute of Business Administration Karachi, Karachi, Sindh, Pakistan; 2 Research Centre for Healthcare and Communities, Coventry University, Coventry, England, UK; 3 Faculty of Health and Wellbeing, Sheffield Hallam University, Sheffield, England, UK; 4 Respiratory Medicine Department, Centre of Exercise and Rehabilitation Science, Leicester Biomedical Research Centre—Respiratory, Glenfield Hospital, Leicester, England, UK; 5 College of Health, Medicine and Life Sciences, Brunel University London, London, UK; 6 Harefield Respiratory Research Group, Guy’s and St Thomas’ Hospitals NHS Trust, London, England, UK; 7 Faculty of Life Sciences and Medicine, King’s College London, London, UK; 8 Centre for Exercise and Rehabilitation Science, NIHR Leicester Biomedical Research Centre, Leicester, England, UK; 9 Centre for Exercise and Rehabilitation Science, University Hospitals of Leicester NHS Trust, Leicester, England, UK

**Keywords:** Pulmonary Disease, Chronic Obstructive, Health Economist, Pulmonary Rehabilitation, Telemedicine, Patient Outcome Assessment, COVID-19, COPD Exacerbations, Compliance

## Abstract

**Introduction:**

Pulmonary rehabilitation (PR) is an effective intervention for patients with chronic obstructive pulmonary disease (COPD) but impact typically only lasts 6–12 months. This paper presents results of an economic evaluation of a PR maintenance programme (Self-management Programme of Activity, Coping and Education (SPACE)) undertaken within a prospective assessor-blind randomised controlled trial.

**Methods:**

Adults with COPD who had completed PR within the previous 4 weeks were randomised to SPACE or best usual care. Healthcare use, personal expenditure and societal costs were recorded at baseline, 6 and 12 months. SPACE costs included staff training, materials and delivery of group sessions. Health utility recorded (EQ-5D-5L) with analysis comparing differences in mean values at 6 and 12 months, over baseline utility scores. Observed changes compared with threshold for COPD clinical significance. Incremental cost-effectiveness ratios estimated from National Health Service and societal perspectives. Cost per quality-adjusted life-year (QALY) values compared with willingness-to-pay threshold (≤£30 000). Uncertainties in costs and outcomes incorporated into a sensitivity analysis. Missing values imputed using a Bayesian mixed model with confounders.

**Results:**

116 patients recruited between October 2019 and June 2022 (57 intervention and 59 control). No significant differences at baseline in age, body mass index, smoking, forced expiratory volume in 1 s and health utility (EQ-5D-5L). Mean healthcare costs in the SPACE group were £139.72 lower per patient over 12 months compared with usual care. At 12 months, the SPACE group retained higher (p=0.04) utility value 0.7609 (SE=0.0238) versus control patients 0.6738 (SE=0.0348). The recorded 0.1178 advantage in mean QALY values (p<0.05) is above the threshold (0.051) for COPD significance. Cost-effectiveness acceptability curves indicate a 97% chance of achieving £20 000 per QALY. Patient and societal costs increase this percentage.

**Discussion:**

This study addresses an important gap in current evidence for non-pharmacological COPD interventions. The PR maintenance programme (SPACE) is shown to be highly cost-effective at 12 months. Future research should consider cost-effectiveness of telerehabilitation programmes, as well as tailored digital support beyond 12 months.

**WHAT IS ALREADY KNOWN ON THIS TOPIC**

- The impact of pulmonary rehabilitation (PR) programmes for patients with chronic obstructive pulmonary disease typically only lasts for 6–12 months once a programme ends.
- PR maintenance programmes are being developed to extend this initial impact, but there is currently no evidence of cost-effectiveness.

**WHAT THIS STUDY ADDS**

- A PR maintenance programme, including a home-based self-management manual and group-based sessions, is shown to be acceptable to patients.
- PR maintenance is cost-effective, producing clinically significant differences in patients’ health-related quality of life and meeting the National Health Service willingness-to-pay threshold.

**HOW THIS STUDY MIGHT AFFECT RESEARCH, PRACTICE OR POLICY**

- These results indicate that a self-management maintenance programme, using behavioural change principles, should now be considered following PR.

## Introduction

 Chronic obstructive pulmonary disease (COPD) is the third leading cause of death worldwide, causing 3.23 million deaths in 2019; and the seventh leading cause of poor health measured by disability-adjusted life years.^1^ This common, progressive respiratory condition is linked to risk factors such as smoking and pollution, with major sequelae impacting on the patient’s longer-term quality of life while they remain alive.^2 3^ As a result, COPD places a large economic burden on society in terms of both direct and indirect costs.^4^ The direct costs of this chronic disease account for 56% (€38.6 billion) of the total cost of respiratory diseases in Europe.^5^ COPD is also a growing healthcare issue with the number of cases globally predicted to increase by 23% over 30 years, approaching 600 million patients globally by 2050.^6^ As a result, COPD costs are projected to increase significantly, reaching a total of US$800 billion over 20 years in the USA.^7^

To date, health technology assessment of COPD therapies has primarily focused on the cost-effectiveness of prescribed medication or oxygen therapy,^8 9^ with self-management interventions designed to maintain health-related quality of life (HRQoL) receiving less attention. Because COPD is not curable, mitigating factors that affect HRQoL are important. Patients experience a variety of pulmonary and extrapulmonary symptoms,^10^ at the same time as their condition continues to deteriorate, even with pharmacological treatments.^11^ The combination of various symptoms, combined with disease progression, significantly affects the HRQoL of patients.^12^,^14^ People living with COPD (plwCOPD) report that symptomatic relief, with the impact this has on their daily lives, is one of the most important aspects of disease management.^15^

For COPD, like other chronic conditions, the clinical trajectory and trajectory-based care is increasingly recognised as being important.^16^ Pulmonary rehabilitation (PR) has been developed as a non-pharmacological intervention to influence a patient’s disease trajectory through an interdisciplinary team approach.^17 18^ PR aims to improve the physical and psychological well-being of patients through tailored therapies (including exercise training, education and behaviour change) specifically designed to promote health-enhancing behaviours.^19^ Clinical guidelines now recommend PR to help manage respiratory symptoms in stable COPD or following a hospital admission for acute exacerbation of COPD (AECOPD).^20^,^22^ A home-based alternative to traditional hospital-based PR has demonstrated similar outcomes at 6 months.^23^,^25^

However, the impact of PR typically only lasts for 6–12 months after the programme ends.^26 27^ A number of self-management maintenance programmes which aim to sustain the initial impact and extend the resulting benefits have been described in the literature.^28 29^ However, a Cochrane review published in 2021 concluded that, although supervised PR maintenance programmes are safe, the evidence on benefits was of low to moderate certainty due to a high risk of bias and small sample sizes.^30^ There is no evidence of the cost-effectiveness of PR maintenance. The most recent American Thoracic Society (ATS) guidelines only include a conditional recommendation for supervised PR maintenance following initial PR, due to low-quality trial evidence.^19^ Similarly, the British Thoracic Society (BTS) concludes that there is insufficient evidence to support routine PR maintenance programmes, while recommending provision of information on self-management after PR completion.^20^ Furthermore, both the ATS and BTS indicate that where resources are limited, healthcare providers should prioritise investment in the initial PR programmes until further evidence on the value of PR maintenance is available.^19 20^

## Methods

### Overview

The research aimed to address the evidence gap on PR maintenance through a randomised controlled trial (RCT) with an integral economic evaluation with the study protocol published in advance.^31^ The specific economic objective was to assess whether a self-management maintenance programme following PR is cost-effective for patients with COPD. A further goal was to strengthen the economic evidence base on self-management for bodies such as the National Institute for Health and Care Excellence (NICE).^21^ This is especially relevant at a time when there are calls to develop cost-effective and safe telerehabilitation programmes to complement centre-based PR.^32^

### Health economic analysis

An integral economic analysis was built into a prospective, two-centre, investigator-blinded, RCT of patients completing PR. The health economic analysis plan was designed to identify whether active self-management by plwCOPD following completion of PR therapy is both effective and cost-effective at 12 months; longer follow-up was not feasible due to funding constraints.^31^ The main cost-utility analysis was undertaken from a healthcare or National Health Service (NHS) perspective, although broader societal and patient-borne costs were also examined. A trial sample size of 116 was estimated based on previous endurance shuttle walk test (ESWT) results.^24^ Although the study presents UK data, the findings should be of interest worldwide.

### Interventions

The Self-management Programme of Activity, Coping and Education (SPACE) for COPD programme, co-designed by patients and healthcare professionals, was developed and pilot tested in Leicester, UK.^33^ The programme consists of a manual for self-management supported by linked facilitated group sessions. Programme facilitators undergo training in delivering the programme using a motivational interviewing approach. The programme has been tested in primary and secondary settings as a home PR alternative compared with usual care^24^ and traditional (centre-based) PR.^23^ More recently, the SPACE programme has been further co-developed with patients with COPD as a maintenance programme following centre-based PR; it had been previously evaluated for PR in primary care and hospital settings.^31^

This programme comprises a 4-stage manual for self-management supported by four linked 2-hour group sessions. These group sessions adopt motivational interviewing techniques alongside the manual. For the present study, group sessions were programmed at months 1, 4, 7 and 10 following completion of PR and included 5–10 participants and two trained facilitators (for further details see online supplemental material 1). The control group received best usual care, consisting of written maintenance advice as recommended by the BTS quality standards.^34^ In addition, both control and intervention group participants were offered referral to a community exercise scheme on PR completion as this represents best usual care in the UK.^35^

### Setting and study population

The trial took place at two UK centres, the University Hospitals of Leicester NHS Trust in Leicester, and Guy’s and St Thomas’ NHS Foundation Trust, Harefield, London. Recruitment to the trial took place between October 2019 and June 2022.

Patients recruited to the study were adults who had completed face-to-face PR within the previous 4 weeks to a standard defined by the BTS.^34^ Individuals were excluded if they were unable to undertake an exercise regime due to significant disability or unable to read English to the reading age of an 8-year-old.

### Measurement of outcomes and costs

#### Outcomes

EQ-5D-5L was selected as the economic outcome measure. Participants completed the questionnaire at baseline, 6 months and 12 months postrandomisation; health is recorded in five dimensions: ‘mobility’, ‘self-care’, ‘usual activities’, ‘pain/discomfort’ and ‘anxiety/depression’ with a total of 3125 health states generated by the EQ-5D descriptive system.^36 37^ Using the value sets for England,^38^ the tariff was applied to each set of responses to the five dimensions to generate an EQ-5D utility score. Analysis of covariance was used to compare differences in mean values for participants in the control and intervention groups at two points in time, that is, 6 and 12 months controlling for baseline value. In addition, any net change was compared with the threshold (0.051) for COPD clinical significance.^39^

A second measurement was also recorded; the self-rated Visual Analogue Scale (VAS) with health state ranging from 100 (best imaginable health state) to 0 (worst imaginable health state).

#### Intervention cost

Bottom-up costing of the PR maintenance programme included: staff training; production of exercise manuals; intervention fidelity checks; and delivery of group sessions (to include staff, room booking, materials and consumables). Unit costs were estimated from trial site records. Session attendance and entries in the exercise manual were used to measure adherence to the home-based maintenance programme. Due to the COVID-19 pandemic, the mode of intervention delivery included telephone/virtual sessions as well as face-to-face group sessions as originally planned. The economic analysis used the higher cost of face-to-face intervention delivery rather than online or hybrid delivery.

#### Healthcare, patient-borne and societal costs

Structured questionnaires (see online supplemental material 2) were used to collect information on NHS resource use, patient out-of-pocket expenditure and other societal costs at baseline, 6 months and 12 months; these included: general practitioner (GP) consultations, practice nurse appointments, psychologist counselling, laboratory investigations, hospital outpatient appointments, inpatient admissions and emergency department visits. Costs were classified as respiratory or non-respiratory. Low frequency health service items were collated under ‘Other costs’ and not reported individually. Medication use was collected to include antibiotics, steroids and respiratory medication. Prescription costs were classified as free (NHS cost) or patient-borne payment.^40^ Referral to community exercise schemes was recorded. Unit costs were extracted from the Health and Social Care Unit Costs manual 2022, and previous studies where needed.^41^,^43^ Pricing using different sources was performed in line with best practice.^44^ Discount rates were not applied due to the 12-month timeline of this study.

### Data analysis

Data were analysed in SPSS V.26 and R V.4.3.1 on an intention-to-treat basis. Missing values were imputed using a Bayesian Mixed Model with confounders that directly or indirectly influenced the HRQoL of the participants. A generalised linear mixed model was fitted both with and without imputation,^45^ for the range of dependent variables, for example, health utilities derived from EQ-5D-5L, GP consultations (see online supplemental material 3, tables A–C for full list). The model adjusted for five covariates (treatment group, time point (baseline, 6 and 12 months), site, age and gender) plus four confounders (body mass index classification, number of chronic conditions, number of medications, smoking pack-years and Medical Research Council (MRC) breathlessness score). An interaction term Time point*Treatment group was incorporated into the model, and a random intercept for each subject was also included.

### Cost-effectiveness and cost-utility analysis

Quality-adjusted life-years (QALYs) were calculated by multiplying the duration of time spent in various health states by the HRQoL weight (ie, utility score for the English general population) associated with that health state, or the simple area-under-the-curve method for incremental QALY gains.^46^ Incremental cost-effectiveness ratios (ICERs) were calculated for intervention over control based on the difference in cost and incremental QALY gains. An incremental net monetary benefit figure was also calculated based on the lower NICE cost-effectiveness threshold of £20 000 per QALY.^47^ Uncertainties in cost and outcomes data were incorporated into a sensitivity analysis using a bootstrapping (non-parametric) technique based on 1000 bootstrap replications to estimate confidence in the observed results from an NHS and societal perspective.^48^,^50^ Cost-effectiveness acceptability curves (CEACs) were constructed to estimate the probability that PR maintenance therapy is cost-effective at different thresholds of decision makers’ willingness to pay for a QALY. These were compared with the NICE acceptance range of £20 000–30 000 per QALY.^47^

### Patient and public involvement

Patients with COPD who were registered with patient and public involvement and engagement (PPIE) groups at the two study sites were invited to review the protocol and two (drawn from Leicester and Harefield London) were involved throughout. As members of the trial steering committee, they contributed to the study design and ensured that patient and public values were reflected in all decision making about research materials and processes in the trial, including participant facing communications, recruitment strategies, interpretation of study results and dissemination of findings. The National Institute for Health and Care Research’s standards for PPIE underpinned all trial processes.^51^

## Results

### Recruitment, intervention completion and data analysis

116 participants were recruited and randomised 1:1 to the PR maintenance programme (n=57) or to usual care (n=59) from 677 patient assessed for eligibility. Intervention completion (defined as at least three quarters of sessions attended) was high (83%). Full content was delivered in 100% of sessions where fidelity checks took place. Control and intervention groups did not differ in terms of their mean HRQoL across trial sites, patient gender, age group, employment, education, living arrangements or participant’s responsibilities, although there was a difference by house type (see online supplemental material 3, table A). Only site, age and gender influenced baseline HRQoL. Two deaths were reported at 12-month follow-up; for these cases, all domains were considered as at level 4 and imputed index and VAS scores valued as zero.

### Health-related quality of life

The mean EQ-5D5L index value for intervention and usual care at baseline, 6 months and 12 months is presented in table 1. Values did not differ significantly between trial groups at baseline and 6 months. However, at 12 months the intervention group retained a significantly higher (p=0.04) utility value 0.7609 (SE=0.0238) versus control patients 0.6738 (SE=0.0348). This produced a significantly greater (p<0.05) decrease in health utility over the 12-month period for the usual care group than that observed for the intervention group −0.0871 (95% CI −0.1712 to −0.0029). Baseline EQ-5D utility score was found to be positively associated with EQ-5D utility score at 6 and 12 months. EQ-5D at 6 and 12 months was found not to be associated with group (control vs intervention).

**Table 1:** Mean health utilities derived from EQ-5D-5L

	Number of patients		Mean quantity (SE)*		Mean treatment difference (Usual care minus SPACE) (95% CI)
	Usual care	SPACE	Usual care	SPACE	
Baseline	59	57	0.7434 (0.0274)	0.7964 (0.0240)	−0.0530 (−0.1253 to 0.0194)
6 months	59	57	0.7061 (0.0291)	0.7538 (0.0226)	−0.0476 (−0.1209 to 0.0257)
Change 0–6 months	59	57	−0.0373 (0.0225)	−0.0426 (0.0213)	NA
12 months	59	57	0.6738 (0.0348)	0.7609 (0.0238)	−0.0871 (−0.1712 to −0.0029)
Change 6–12 months	59	57	−0.0323 (0.0276)	0.0071 (0.0198)	NA
Change 0–12 months	59	57	−0.0696 (0.0280)	−0.0355 (0.0206)	NA

* Missing values replaced using Bayesian linear mixed model, taking into account covariates and confounders.

NA, not assessed; SPACE, Self-management Programme of Activity, Coping and Education; VAS, visual analogue scale.

Self-rated quality of life, measured through the VAS, demonstrates a similar pattern.

Closer analysis of the principal EQ-5D components identified that most participants reported problems in all five domains. At baseline, fewer than 14% reported ‘no problem’ in all domains; at 12 months this figure was 6.8% (controls) and 12.3% (intervention group). Mean health utility values were also analysed over the COVID-19 pandemic period. Although health utility values were highest in the pre-COVID period, followed by during COVID and lowest in the post-COVID period, mean values did not differ significantly between COVID periods or across the two trial groups.

### NHS costs

The estimated overall cost of PR maintenance was £18 293.80 for the 57 patients recruited or £320.93 per patient. Within this, the delivery of group sessions (face-to-face as originally planned) was estimated to cost £699.84 to £814.24 per session for 5–10 participants. A mean cost per patient was estimated based on reported NHS service utilisation and relevant unit costs (see online supplemental material 3, tables C and D). An overview of costs over the trial period is presented in table 2, together with CIs. Use of NHS services (ie, primary care, outpatient, Emergency Department visits, hospital inpatient stay) shows a lower mean value of £1309.44 (SE=£301.71) for the intervention group, compared with £1441.25 (SE=£305.70) for control patients, over 12 months. This difference was primarily driven by reduced numbers of GP visits in the intervention group. In terms of annual NHS prescription costs, the mean value was £877.50 per patient (>90% receive free prescriptions) with a higher amount for usual care by £75.22 (95% CI £96.77 to £247.21), although the difference was non-significant (p>0.10).

**Table 2:** Mean NHS costs (£) of healthcare resource use over 12 months

Type of healthcare resource use	Number of patients		Mean cost (SE)*		Mean cost difference (£) (Usual care minus SPACE) (95% CI)
	Usual care	SPACE	Usual care	SPACE	
NHS services					
GP consultations	59	57	175.00 (27.70)	87.19 (11.09)	87.81 (27.94 to 147.68)
Practice nurse	59	57	30.91 (8.15)	24.99 (7.67)	5.92 (−16.28 to 28.13)
Psychologist/counsellor	59	57	19.01 (13.73)	17.37 (12.23)	1.65 (−34.86 to 38.15)
Pulmonary services	59	57	340.43 (143.67)	208.81 (108.18)	131.62 (−226.48 to 489.71)
Inpatient short stay	59	57	14.32 (14.31)	44.46 (25.21)	−30.14 (−87.08 to 26.79)
Inpatient long stay	59	57	507.81 (181.73)	375.45 (194.05)	132.36 (−393.87 to 658.59)
Emergency Department visit	59	57	185.49 (85.71)	80.00 (24.69)	105.49 (−73.84 to 284.82)
Others†	59	57	174.01 (78.20)	89.95 (31.45)	84.06 (−85.05 to 253.18)
Total NHS services‡	59	57	1441.25 (305.70)	1309.44 (301.71)	131.80 (−719.51 to 983.13)
Prescriptions (free to patients)					
Respiratory	50	55	245.70 (25.79)	286.03 (22.91)	−22.33 (−90.51 to 45.84)
Antibiotics	50	55	71.37 (14.11)	73.39 (14.66)	−2.02 (−42.54 to 38.50)
Steroids	50	55	42.12 (9.61)	51.05 (2.24)	−8.93 (−40.22 to 22.35)
All prescriptions (free cases)	50	55	941.85 (79.74)	819.00 (50.14)	122.85 (−60.47 to 306.17)
Prescriptions cost for all cases	59	57	898.32 (71.12)	823.11 (48.81)	75.22 (−96.77 to 247.21)
Healthcare costs (without intervention delivery cost)	59	57	2239.41 (317.53)	1778.77 (300.98)	460.63 (−407.14 to 1328.41)
Total healthcare costs	59	57	2239.41 (317.53)	2099.70 (300.98)	139.72 (−728.14 to 1007.41)

* Missing values replaced using Bayesian linear mixed model, taking into account covariates and confounders.

† Others include a long list of various services; this aggregate cost was determined after micro-costing individual cases.

‡ Total NHS cost also includes the cost towards diagnostic procedures including blood tests provided by the NHS.

GP, general practitioner; NHS, National Health Service; SPACE, Self-management Programme of Activity, Coping and Education.

The overall NHS service cost per participant over 12 months was lower in the PR maintenance group at £1778.77 (SE=£300.98) compared with £2239.41 (SE=£317.53) for controls. The mean between-group difference in costs was £460.63 (95% CI −£407.14 to £1328.41). Once the cost of the PR maintenance programme is taken into account, the estimated between-group cost advantage for the intervention group falls to £139.72 per patient (95% CI −£728.14 to £1007.41).

### Patient-borne and societal costs

A summary of patient out-of-pocket and societal expenditure over the trial period is presented in table 3. Patients’ mean out-of-pocket expenditure was estimated to be £137.56 (SE=£36.26) for the intervention group, and £270.48 (SE=£57.17) for control patients. Use of council-funded community exercise programmes was higher for the intervention group at £35.44 (SE=£19.4) than for controls at £13.90 (SE=£8.73). In terms of wider societal costs, recovery can increase economic activity, reduce social security benefit payments and loss of tax revenues generated through work. There were no statistically significant differences in employment status between groups at baseline and 12 months. Only a small minority (one-fifth) of participants were in work or self-employed.

**Table 3:** Mean patient-borne costs and societal expenditure (£) over 12-month period

Type of expenditure	Number of patients		Mean cost (SE)*		Mean cost difference (£) (Usual care minus SPACE) (95% CI)
	Usual care	SPACE	Usual care	SPACE	
Patient-borne (out-of-pocket)					
Personal cost	59	57	170.34 (47.42)	104.71 (28.71)	65.62 (−45.11 to 176.36)
Paid medicine prescription cost	59	57	100.14 (35.84)	32.84 (23.75)	67.30 (−8.46 to 153.07)
Total patient-borne costs	59	57	270.48 (57.17)	137.56 (36.26)	132.93 (−2.22 to 268.07)
Other					
Community Exercise Scheme†	59	57	13.90 (8.73)	35.44 (19.4)	−21.54 (−63.21 to 20.13)
Total cost societal perspective‡	59	57	2523.81 (325.31)	2272.70 (313.05)	251.11 (−644.12 to 1146.34)

* Missing values replaced using Bayesian linear mixed model, taking into account covariates and confounders.

† Local authority funded Community Exercise Scheme.

‡ Includes NHS costs and SPACE Intervention delivery, patient-borne costs and local authority expenditure.

NHS, National Health Service; SPACE, Self-management Programme of Activity, Coping and Education.

Once broader societal and patient-borne costs are added to NHS costs, the mean between-group difference in total cost per participant rises from £139.72 to £251.11 (95% CI −£644.12 to £1,146.34).

### Cost-effectiveness

Table 4 presents incremental cost-effectiveness analyses from both the NHS perspective and a broader perspective, including societal and patient-borne costs. From the NHS perspective, healthcare resource use with the intervention was approximately £139.72 lower per patient than the mean cost of usual care; and there was an incremental QALY gain over the 12-month period of 0.1178 (p<0.05) for intervention patients over patients in the usual care group.

**Table 4:** ICERs and INMB from NHS and societal perspectives

Cost/QALY per participant	NHS perspective		Societal perspective	
	SPACE (n=57)	Usual care (n=59)	SPACE (n=57)	Usual care (n=59)
1. Cost of SPACE intervention	£18 293.80	£0.00	£18 293.80	£0.00
2. NHS healthcare use cost	£56 344.26	£85 033.62	£56 344.26	£85 033.62
3. Medication (free prescription) cost to NHS	£45 045.00	£47 092.50	£45 045.00	£47 092.50
4. Personal cost+paid medication	NA	NA	£7840.61	£15 958.39
5. Community Exercise Scheme	NA	NA	£2020.08	£820.10
Total costs (1–4)	£119 683.06	£132 126.12	£129 543.75	£148 904.61
All costs per participant	**£2099.70**	**£2239.43**	**£2272.70**	**£2523.81**
QALY—Baseline	0.7964	0.7434	0.7964	0.7434
QALY—6 months	0.7538	0.7061	0.7538	0.7061
QALY—12 months	0.7609*	0.6738*	0.7609*	0.6738*
QALY difference 6 months over base	−0.0426†	−0.0373	−0.0426†	−0.0373
QALY difference 12 months over base	−0.0355	−0.0696†	−0.0355	−0.0696†
Incremental QALY gains‡	I over C§	0.1178	I over C	0.1178
Difference in costs	I over C	−£139.72	I over C	−£251.11
ICER	I over C	−£1186.61	I over C	Dominant
INMB¶	I over C	−£139.72	£2356	£2607.11

* Significant difference between groups at p<0.05.

† QALY change significant at p<0.05.

‡ Incremental QALYs, cost-utility analysis estimated by the area-under-the-curve for intervention and usual care patients.

§ SPACE intervention (I) over usual care (C).

¶ Incremental net monetary benefit estimate employing the NHS cost-effectiveness threshold of £20 000 per QALY.

ICERs, incremental cost-effectiveness ratios; INMB, incremental net monetary benefit; NA, not assessed; NHS, National Health Service; QALY, quality-adjusted life-year; SPACE, Self-management Programme of Activity, Coping and Education.

The mean QALY difference of 0.1178 is well above the threshold of 0.051 judged to represent a significant clinical impact in COPD.^39^ Because the intervention is both less expensive and more effective than usual practice, a negative ICER value indicates ‘dominance’ for PR maintenance from the NHS perspective. Table 4 also presents the incremental net monetary benefit (INMB) based on the NHS cost-effectiveness threshold of £20 000 per QALY. This similarly indicates that the intervention is cost-effective at the lower NICE accepted threshold.

**Figure 1:**
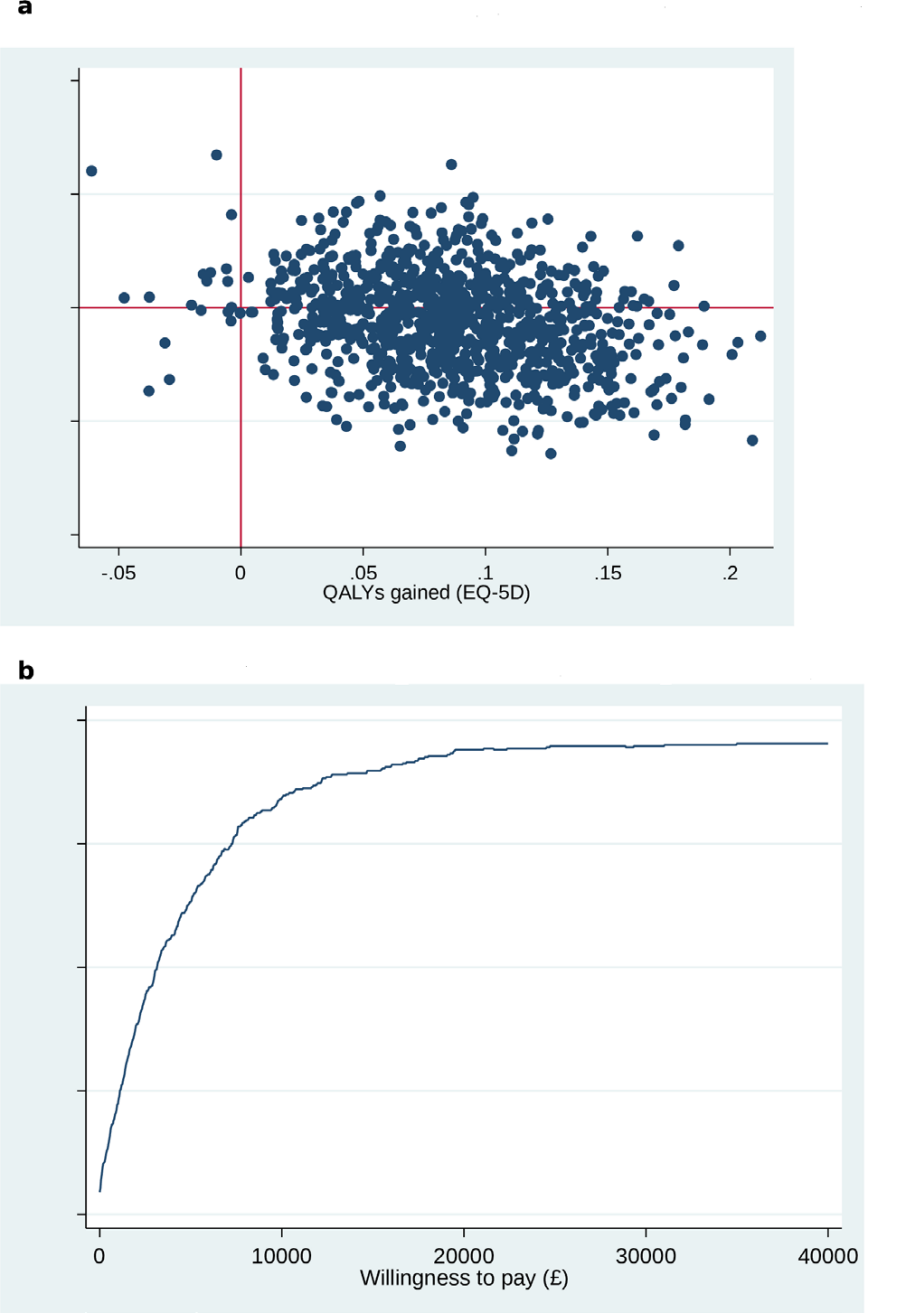
Cost-effectiveness plane and CEAC. (a) Cost-effectiveness plane (NHS perspective), bootstrap samples using GLM model. (b) CEAC (NHS perspective). CEAC, cost-effectiveness acceptability curve; EQ-5D, 5-level EQ-5D-5L version GLM, generalised linear mixed; NHS, National Health Service; QALY, quality-adjusted life-year.

Uncertainty in these findings was estimated using a bootstrapping technique.^48^ Figure 1a, based on 1000 bootstrap estimates from an NHS perspective, shows that the majority of points in the cost-effectiveness plane lie to the right of the vertical line, clearly confirming QALY gain for PR maintenance. Incremental cost points straddle the horizontal line (zero) indicating some uncertainty about the size of cost-saving; however, most points (60%) lie in the dominant south-east quadrant.

The CEAC in figure 1b indicates the probability that PR maintenance is cost-effective at different willingness to pay thresholds. This confirms that there is a 97% chance the intervention will be cost-effective at a threshold of £20 000 per QALY, and 90% at the more ambitious threshold of £10 000 per QALY; the current NICE acceptance level ranges from £20 000 to £30 000 per QALY.^47^ Inclusion of broader societal and patient costs raises the former probability above 97% and increases the number of bootstrap points in the dominant south-east quadrant of the cost-effectiveness plane. Figures incorporating a broader societal perspective are presented in online supplemental material 4.

## Discussion

### Principal findings

To our knowledge, this is the first analysis of the cost-effectiveness of a self-maintenance programme for plwCOPD following completion of centre-based PR. Our findings show that the ‘SPACEforCOPD’ programme co-developed with patients and tested over 10 years in other settings was highly cost-effective in extending the benefits of PR and reducing NHS costs by £139.72 per patient (95% CI −£728.14 to £1007.41). When scaled to a typical programme size of 10 individuals, the expected gain in QALYs is 1.178 with net savings averaging £1397.20. Individuals supported to self-manage their chronic condition therefore appear able to maintain their own HRQoL and, as a consequence, are better able to use healthcare resources. The cost-effectiveness plane (1000 bootstrap estimates) shows a clear impact in terms of HRQoL being maintained. In terms of cost-effectiveness, the CEAC indicates a 97% chance that the intervention is cost-effective at £20 000 per QALY, the lower acceptance threshold in the £20 000–30 000 range. This value is based on differential outcomes at 12 months and may improve further if the differential impact of the PR maintenance programme is sustained beyond 1 year.^52^ compared with willingness-to-pay threshold levels globally, the intervention value of £20 000 or US$25 929 per QALY is well within the levels set in the USA (US$50 000 per QALY), Australia ($A69 000 or US$43 700 per QALY), Ireland (€45 000 or US$48 970 per QALY), but above the threshold level in Malaysia (MYR29 080 or US$6555 per QALY).^53^

The present economic evaluation measured effectiveness using EQ-5D-5L which enabled calculation of incremental QALY gains.^37^ To date, much of the evidence base for COPD interventions relies on clinical outcome measures, including a 2024 economic evaluation of telerehabilitation to complement centre-based PR for people with chronic respiratory disease.^54^ The present trial demonstrated no statistically significant difference between groups at 12 months for the primary clinical outcome, the ESWT, expressed in seconds, showing endurance exercise tolerance maintained in both groups.^55^ However, COPD is a complex disease and, although it primarily affects the lungs, it has multiple extrapulmonary symptoms that will have a high impact on the daily burden of the disease.^10 12 13 56 57^ Overall health utility may therefore only be partially represented by disease-specific measures. A recent systematic review of outcome measures used to evaluate PR identified some studies including EQ-5D as an outcome, but none used this to calculate incremental QALYs.^58^ In the present study, when we compared the utility gain at 1 year with that judged to be clinically significant for COPD, we found this reached well above the threshold for clinical significance.^39^

### Comparison with other studies

As well as a concentration on physiological outcomes such as exercise capacity,^2259^,^62^ to date there is little evidence on the comparable effectiveness and cost-effectiveness of a range of interventions more broadly. In 2016, the London Respiratory Network presented cost per QALY estimates for different pharmacological and non-pharmacological interventions in the form of a COPD Value Pyramid.^63^ Comparing our findings with other interventions in the Pyramid shows that PR maintenance falls in a similar range to long-term oxygen therapy at £13–18 000 per QALY (2022 prices). Cost-per-QALY bench-mark data for other non-pharmacological interventions are currently lacking for COPD; the BTS Respiratory Futures website mentions only one study, an evaluation of cognitive behavioural therapy to reduce anxiety which reports 100% probability that CBT will be cost-effective (compared with self-help leaflets) at all thresholds above £5000 (€5727).^64 65^ Similarly, the most recent Global Initiative for Chronic Obstructive Lung Disease report includes few mentions of any economic evaluations.^66^ Those mentioned cover other aspects such as: case finding in primary care^67^; screening strategies for identifying undiagnosed COPD^68^; improved inhaler adherence^69^; and nutritional intervention programme.^70^ Finally, although extension of the COPD Value Pyramid to include more interventions has been advocated, at present, these suggestions do not include PR maintenance.^63 71 72^

Separately, an early trial of a PR maintenance intervention, which included an integral economic evaluation, identified a low probability (72.9%) that the intervention would be cost-effective at a threshold of £20 000 per QALY.^73^ In the present study, we recorded a high (97%) probability for the SPACE intervention, and even at the lower threshold of £10 000 per QALY a 90% chance; further, broadening the economic perspective to include patient-borne and societal costs increased these probabilities. This difference is probably due to the intervention being evaluated. The ATS Clinical Practice Guidelines for PR for adults with COPD published in 2023 report that trials are generally heterogeneous in nature and the types of intervention studied.^19^ Only 3 out of 185 references cited in the guidelines include some form of economic evaluation; these focus on PR hospital outreach, home-based PR and early PR following AECOPD.^74^,^76^

### Strengths and limitations of study

The main strength of this study is that the economic evaluation was embedded in a fully powered RCT. The case for considering cost-effectiveness is strengthened by the fact that the trial found no difference between groups at 12 months for the primary clinical outcome measure.^55^ In its turn, the multicentre trial design also ensured that the economic findings are more generalisable than in a single centre study.

One limitation of this study is recruitment of English speakers only, although at both sites all individuals who met the inclusion criteria were approached. Overall, 90% of participants were from a white British background. The national audit reports that 82.5% of patients assessed for PR are white British; for Harefield, London and Leicester, this figure is 90% and 92%, respectively.^77^ A second limitation may be an element of self-selection, resulting in a more motivated research population. Although randomisation ensured that this should not affect differences between groups, more widespread implementation may result in lower adherence and reduced effectiveness. Third, the pandemic may have had an impact. Although comparison of HRQoL in sequential groups (ie, patients recruited pre-COVID, during COVID, post-COVID) revealed highest mean values pre-COVID and lowest post-COVID, differences were not significantly different between COVID periods or across the two trial groups. Similarly, although the pandemic reduced face-to-face data collection, which may have affected completion of questionnaires by some patients, this was addressed by imputation. Use of healthcare services may also have been affected, although recent research suggests that the mode of access (virtual or in-person) rather than uptake level was affected.^78^ Once again, we would expect both trial groups to be similarly affected, while acknowledging that healthcare utilisation levels may have increased or decreased. Finally, the cost of the SPACE intervention was based on face-to-face group sessions as originally planned. In the event, during the pandemic, some sessions were delivered online at a lower cost. This means that the estimate of cost-effectiveness presented here is probably conservative. Furthermore, emerging evidence demonstrates the cost-effectiveness of adding telerehabilitation to centre-based PR for people with chronic respiratory disease.^75^ NICE has already recommended that a digital technology can be used to deliver PR programmes in the NHS while economic evidence on its cost-effectiveness is generated.^79^ The cost-effectiveness of PR maintenance programmes might be further increased by introducing telerehabilitation, incorporating telemonitoring and some synchronous supervision.

### Conclusions and recommendations

This study addresses an important gap in the current evidence base for non-pharmacological COPD interventions. Our research confirms that investment in a maintenance programme following PR can be highly cost-effective. Further follow-up beyond 12 months would enable suggested longer-term cost-effectiveness to be established.^52^ The study also highlights the value of including health utility in the core outcome sets being proposed to improve the evidence base for PR and support future investment decisions.^80^ Further exploration of the relationship between commonly used clinical outcome measures and health-utility would add to the emerging evidence base in this patient group.^81^ Finally, in terms of the intervention itself, additional studies should now examine different modes of delivery such as telerehabilitation, as well as the value of extending tailored digital support beyond 12 months.

## Supplementary material

10.1136/bmjresp-2025-003406 online supplemental file 1

10.1136/bmjresp-2025-003406 online supplemental file 2

10.1136/bmjresp-2025-003406 online supplemental file 3

10.1136/bmjresp-2025-003406 online supplemental file 4

## Data Availability

Data are available upon reasonable request.
