# Supplementary material for: Cost-effectiveness of a self-management maintenance programme following pulmonary rehabilitation: a UK randomised controlled trial for patients with chronic obstructive pulmonary disease
Source: BMJ Open Respir Res. 2025 Dec 4;12(1):e003406. doi: 10.1136/bmjresp-2025-003406 (PMC12684092; doi:10.1136/bmjresp-2025-003406)
Supplement: online supplemental file 1 [file bmjresp-12-1-s001.pdf]

Supplemental Material- Appendix 1

Home-Based Exercise Manual and Group-Based Sessions

A. Home-based exercise manual SPACE FOR COPD©

The manual is divided into four stages. It includes self-management skills, disease specific information and tasks. The content of the manual has been approved by the Plain English Campaign and received the Crystal Mark for Clarity of British English. Table 1 outlines the manual content.

Table 1: Manual components

**Stage 1**<br>What's happened to your lungs?<br>Exercise: How to get fitter<br>Setting your goals<br>Managing your stress - part 1<br>Your emotions<br>Controlling your breathing<br>Information about your medication	**Stage 4**<br>Staying fit and your hobbies<br>Your relationships<br>Dealing with setbacks<br>Sex and your lungs<br>Breathe Easy<br>Conclusion
**Stage 2**<br>How to stay fit<br>Managing days when you feel unwell<br>Saving your energy<br>Diet and feeling unwell<br>Advice for clearing your chest	**FAQs & Appendix**<br>Frequently asked questions<br>Setting your walking speed<br>Help for carers<br>Advice about oxygen<br>Smoking: advice on giving up<br>Cleaning your breathing devices<br>Spare walking diaries<br>Spare strength training diaries
**Stage 3**<br>How to get stronger<br>Managing your stress - part 2<br>Healthy eating<br>Travelling and your lung disease	

- Self-management components of the manual were mapped to the PRISMS taxonomy framework.[1]
- A COPD action plan was inserted consistent with each centres' usual care.
- A single page insert encouraged patients to follow the SMART (Specific, Measurable, Achievable, Relevant, and Time bound) method for goal settings.
- Healthcare Practitioners (HCPs) advised participants on how to use and follow the manual independently at home.
- Participants were asked to complete sections of the manual at home (e.g. exercise diaries) and they had the opportunity to call HCPs with questions via telephone.

B. Group-based maintenance sessions

The PR maintenance programme sessions were held in small groups (5 to 10 participants per group). These accompanied the SPACE for COPD manual with the aim of discussing progress, addressing barriers, increasing motivation to maintenance, enhancing health lifestyle behaviours, and encouraging social support. Facilitators used motivational interviewing techniques to identify barriers and facilitators and support self-efficacy. The manual could be used by participants to refer back to various education topics as required (e.g. insert some of the topics above). Figure 1 shows the content of the 4 sessions. Each group session was facilitated by two HCPs (e.g. Physiotherapists, respiratory specialist nurses, health psychologists). The sessions were delivered according to the facilitator manual and information from the manual was adapted to focus on maintenance and reinforcement rather than repetition of core education learned in PR.

Figure 1: Content of 4 group sessions

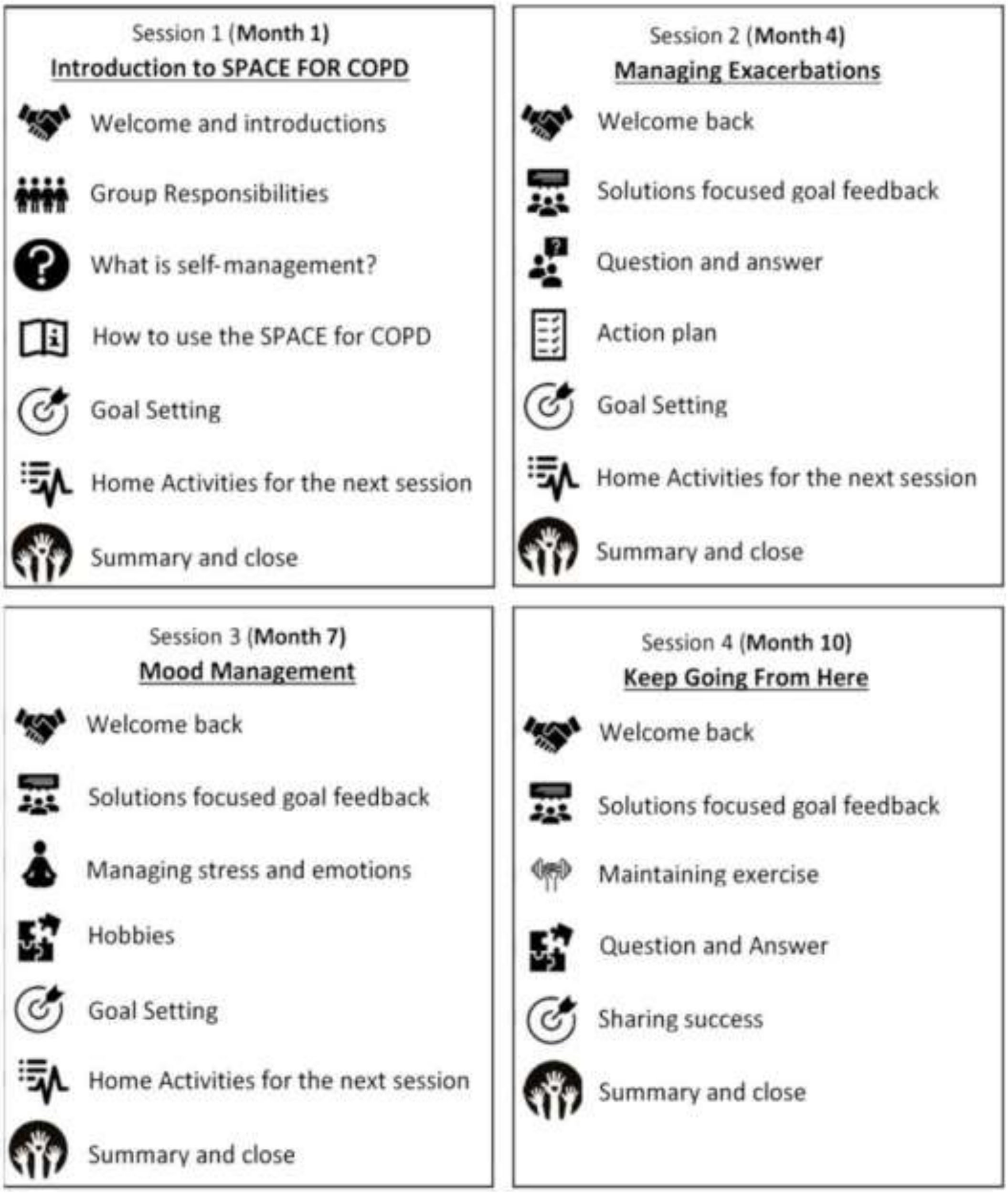

C. Staff training and intervention fidelity

HCPs were trained to deliver the SPACE FOR COPD© maintenance programme by a health psychologist and a specialist nurse. All attended a one-day training course, supplemented with written material and DVD tutorials to take away.

The training day covered: motivational interviewing skills, introduction to the facilitator manual and review of SPACE FOR COPD© manual, practical workshop, group facilitation, learning outcomes re-visited, and trial management. All the HCPs had experience in delivering pulmonary rehabilitation.

A Quality Assurance process assessed delivery of intervention content and educational style.

- Intervention fidelity checklists for facilitators were adapted from a previous study.[2]
- One session per group was peer-observed and the checklist completed.
- Each site observed the opposite site's group. Facilitators completed fidelity checklists at the end of each session.

Reference

1. Pearce, G., et al., *The PRISMS taxonomy of self-management support: derivation of a novel taxonomy and initial testing of its utility.* J Health Serv Res Policy, 2016. **21**(2): p. 73-82.
2. Bourne, C., et al., *A Self-Management Programme of Activity Coping and Education - SPACE for COPD(C) - in primary care: The protocol for a pragmatic trial.* BMJ Open, 2017. **7**(7): p. e014463.
