# Supplementary material for: Cost-effectiveness of a self-management maintenance programme following pulmonary rehabilitation: a UK randomised controlled trial for patients with chronic obstructive pulmonary disease
Source: BMJ Open Respir Res. 2025 Dec 4;12(1):e003406. doi: 10.1136/bmjresp-2025-003406 (PMC12684092; doi:10.1136/bmjresp-2025-003406)
Supplement: online supplemental file 2 [file bmjresp-12-1-s002.pdf]

Supplemental Material Appendix 2: Questionnaires

1. Baseline Questionnaire

Attendance Costs

The following questions are about how much it costs you to attend your hospital/clinic appointment today.

Please tick all the boxes that apply to you.

Travel

1. For this visit to the hospital/clinic how did you travel?

**Own Car**	☐	**Ambulance**	☐
**Taxi**	☐	**Other, please describe**	☐
**Bus / train**	☐	……………………………….	

2. If you travelled by bus, taxi, or train, how much did you pay for return fare (i.e. travel both ways)?

£……………

3. Approximately how many miles from your home is the hospital/clinic?

……….miles

Dependants

4. Do you have any children or other dependants for whom you had to make care arrangements in order to be able to attend the hospital/clinic?

Yes ☐ No ☐

5. If 'Yes', approximately how much did it cost you?

£……………

Time spent by you

6. Did you take time off paid work to attend the hospital/clinic?

Yes ☐ No ☐

7. If 'Yes', how much was your wage loss? (*if none, enter '0'*)

£……………

8. Did someone accompany you for your appointment today?

Yes ☐ No ☐

9. If ‘Yes, did they take time off paid work to attend the hospital/clinic?

Yes ☐ No ☐

10. If ‘Yes’, how much was their wage loss? (*if none, enter ‘0’*)

£..............

Other Costs

11. Were there any other costs for this visit to the hospital/clinic (e.g. parking)?

Yes ☐ No ☐

12. If ‘Yes’, how much were these costs in total?

£..............

Reimbursement

13. Were any of your attendance costs reimbursed by the hospital/clinic/someone else?

Yes ☐ No ☐

14. If ‘Yes’, which costs?

……………………………………………………………………………………..

To assess whether the people taking part in this study are representative of people in the local population, we would like to ask you a few extra questions.

The information given by you will remain confidential and will not be read by anyone else. It will only be used by the research team.

15. What is your current Employment Status?

Employed Full Time ☐
Employed Part Time ☐
Self-employed ☐
Unemployed ☐
Retired/ Student ☐
Other ☐

Thank you for completing this questionnaire

2. Six-Month Questionnaire

Health/ Social Care & Personal Costs

This section is about health care & other services you have received since you started the study six months ago. Please read each question carefully. For each question, if you have had no treatments or personal costs, please enter '0'.

Community Health & Social Care Services

1. In the **last six months**, how often have you used the following **NHS or other services**? (Please do not include any sessions or treatments that you attended as part of the study).

		Number of times
1a	Your GP or another GP (*if none enter '0'*)	☐☐
1b	Practice nurse (*if none enter '0'*)	☐☐
1c	Psychologist/ Counsellor (*if none enter '0'*)	☐☐
1d	Hospital A & E attendance (*if none enter '0'*)	☐☐
1e	Pulmonary Rehabilitation (*if none enter '0'*)	☐☐
1f	Community Exercise Scheme (if *none enter '0'*)	☐☐
1g	Other NHS service (please specify): ______________________	☐☐
1h	Social care service (please specify): ______________________	☐☐
1i	Other service (please specify): ______________________	☐☐

Hospital inpatient stay(s)

2. In the **last six months** have you been admitted to hospital due to your lung condition?

Yes ☐ No ☐

3. If 'Yes', how many days/ hrs were you in hospital? (*if you can't remember enter '0'*)

……….days OR ……….hours

Personal costs.

4. In the **last six months**, have you spent money on things such as walking shoes, activity tracker/fitbit, gym membership, exercise machine, domestic services, complementary therapy or any other products, equipment or services to support **your** physical health? (please list items below & enter cost to nearest pound).

Item (brief description)	Total spent (£)
1.	
2.	
3.	
4.	
5.	

To assess whether people in the study have been able to return to normal activities, we would like to ask you a few extra questions.

The information given by you will remain confidential and will not be read by anyone except the research team.

Employment status

5. What is your current Employment Status?

Employed Full Time ☐
Employed Part Time ☐
Self-employed ☐
Unemployed ☐
Retired/ Student ☐
Other ☐

If you are not in paid employment, please go to question 12

Time off work/Altered working hours

6. In the **last six months** have you had to change your occupation due to your lung condition?

Yes ☐ No ☐

7. If 'Yes', what is your new job? ______________________________

8. In the **last <u>six</u> months** have you taken any days off sick from work due to your lung condition?

Yes ☐ No ☐

9. If 'Yes, how many days in total (*if you can't remember enter '0'*) ________days

10. Have your hours of work altered in the **last <u>six</u> months** due to your lung condition?

Yes decreased ☐ Yes increased ☐ No ☐

11. If 'Yes', by how many hours per week (approximately)? ________hours per week

12. Over the **last <u>six</u> months**, on approximately how many days has your lung condition stopped you undertaking these activities? (*if none enter '0'*)

	Total number of days
a. Education	☐☐
b. Childcare/care of a relative	☐☐
c. Housework e.g. cleaning, food shopping	☐☐
d. Voluntary work	☐☐
e. Other (please specify) ______________________________	☐☐

Thank you for completing this questionnaire

3. Twelve Month Questionnaire

Health/ Social Care & Personal Costs

This section is about health care & other services you have received since you started the study <u>twelve months</u> ago. We are interested in the <u>most recent six months</u>. Please read each question carefully. If you have had no treatments or personal costs, please enter '0'.

Community Health & Social Care Services

1. In the **last <u>six</u> months**, how often have you used the following **NHS or other services**? (Please do not include any sessions or treatments that you attended as part of the study).

		Number of times
1a	Your GP or another GP (*if none enter '0'*)	☐☐
1b	Practice nurse (*if none enter '0'*)	☐☐
1c	Psychologist/ Counsellor (*if none enter '0'*)	☐☐
1d	Hospital A & E attendance (*if none enter '0'*)	☐☐
1e	Pulmonary Rehabilitation (*if none enter '0'*)	☐☐
1f	Community Exercise Scheme (if *none enter '0'*)	☐☐
1g	Other NHS service (please specify): ____________________	☐☐
1h	Social care service (please specify): ____________________	☐☐
1i	Other service (please specify): ____________________	☐☐

Hospital inpatient stay(s)

2. In the **last <u>six</u> months** have you been admitted to hospital due to your lung condition?

Yes ☐ No ☐

3. If 'Yes', how many days/ hrs were you in hospital? (*if you can't remember enter '0')*

……….days OR ……….hours

Personal costs.

4. In the **last <u>six</u> months**, have you spent money on things such as walking shoes, activity tracker/fitbit, gym membership, exercise machine, domestic services, complementary therapy or any other products, equipment or services to support **your** physical health? (please list items below & enter cost to nearest pound).

**Item (brief description)**	**Total spent (£)**
1.	
2.	
3.	
4.	
5.	

To assess whether people in the study have been able to return to normal activities, we would like to ask you a few extra questions.

The information given by you will remain confidential and will not be read by anyone except the research team.

Employment status

5. What is your current Employment Status?

- Employed Full Time ☐
- Employed Part Time ☐
- Self-employed ☐
- Unemployed ☐
- Retired/ Student ☐
- Other ☐

If you are not in paid employment, please go to question 12

Time off work/Altered working hours

6. In the **last <u>six</u> months** have you had to change your occupation due to your lung condition?

Yes ☐ No ☐

7. If 'Yes', what is your new job? ____

8. In the **last <u>six</u> months** have you taken any days off sick from work due to your lung condition?

Yes ☐ No ☐

9. If 'Yes, how many days in total (*if you can't remember enter '0'*) ________days

10. Have your hours of work altered in the **last <u>six</u> months** due to your lung condition?

Yes decreased ☐ Yes increased ☐ No ☐

11. If 'Yes', by how many hours per week (approximately)? ________hours per week

12. Over the **last <u>six</u> months**, on approximately how many days has your lung condition stopped you undertaking these activities? (*if none enter '0'*)

	Total number of days
a. Education	☐☐
b. Childcare/care of a relative	☐☐
c. Housework e.g. cleaning, food shopping	☐☐
d. Voluntary work	☐☐
e. Other (please specify) ______________________________	☐☐

Thank you for completing this questionnaire
