# Supplementary material for: Cost-effectiveness of a self-management maintenance programme following pulmonary rehabilitation: a UK randomised controlled trial for patients with chronic obstructive pulmonary disease
Source: BMJ Open Respir Res. 2025 Dec 4;12(1):e003406. doi: 10.1136/bmjresp-2025-003406 (PMC12684092; doi:10.1136/bmjresp-2025-003406)
Supplement: online supplemental file 3 [file bmjresp-12-1-s003.pdf]

Supplemental Material Appendix-3

Demographics, Confounders & NHS Service Utilisation

Table 1: Confounders: Demographics, Social and Chronic Conditions at Baseline by Group

Confounder/Covariate		*Group A (Control)*	*Group B (Intervnt)*	*Mean Difference*	*F- or t-value*	*p value*
Gender (%)	Male	50.8	64.9		.273	.602
	Female	49.2	35.1			
Age Tertile (%)	Up to 65	30.5	35.1		.326	.569
	66-75	44.1**	26.3**			
	76-99	25.4	38.6			
Mean Age (years)		69.5	71.0	-1.492	-.860	.391
House Type	Bungalow	11.9	24.6		2.014	.159
	Flat	13.6	8.8			
	House	74.6	66.7			
% having stairs		83.1	71.9		2.063	.154
Living Arrangements	Alone	27.1	38.6		2.570	.112
	With Spouse	61.0	45.6			
	With Family/Other	11.9	15.8			
Responsibilities for	None	61.0	52.6		3.671	.058
	Pets	28.8	17.5			
	Carers/Grandchildren	10.2**	29.8**			
Employment status	Full-time	8.5	6.8		.667	.416
	Part-time	6.8	5.3			
	Self-employed	5.1	7.0			
	Unemployed/can't work	5.1	12.3			
	Retired	74.6	66.7			
% Working		20.4	19.1		.034	.854
Education	Up to Secondary	89.8	87.7		.128	.721
	Above Secondary	10.2	12.3			
BMI classification (%)	Underweight/Missing	10.2	10.5		.281	.597
	Healthy	28.8	21.1			
	Overweight	25.4	29.8			
	Obese	35.6	38.6			
Smoking - Pack Years Tertile (%)	Never Smoked	6.8	5.3		.111	.740
	up to 30	28.8	35.1			
	31-45	33.9	31.6			
	46-149	30.5	28.1			
Mean Pack Years		41.5	39.0	2.499	.498	.620
Other Chronic Conditions Reported (%)	1. Respiratory	25.4	21.1		.306	.581
	2. Cardiac	49.2	49.1		.000	.997
	3. Rheumatism	37.3	29.8		.716	.399
	4. Gastrointestinal	16.9	14.0		.185	.668
	5. Cancer	13.6	22.8		1.667	.199
All Other Chronic		82.4	86.0		1.068	.304
No. of Other Chronic Conditions (%)	0 (COPD only)	13.6	14.0		.336	.563
	1	23.7	17.5			
	2	22.0	35.1			
	3-4	25.4	26.3			
	5+	15.3	7.0			
Mean Chronic Conditions		2.4	2.1	0.318	1.033	.304
Medications Tertile (%)	1-5	37.3	29.8		0.000	1.000
	6-8	25.4	40.4			
	9-25	37.3	29.8			
Mean No. of Medications		7.4	6.9	0.494	.663	.508

** Proportions differ significantly at 5% level between groups

Table 2: Significant Confounders with Mean HRQoL at Baseline

		Mean	SE of Mean	F-value/ significance/eta square
BMI classification (%)	Underweight/Missing	.7193	.0552	F=3.415 p=.020 eta sq.=.084
	Healthy	.8207	.0252	
	Overweight	.8259	.0270	
	Obese	.7069	.0370	
Smoking - Pack Years Tertile (%)	Never Smoked	.6854	.1194	F=1.026 p=.384 eta sq.=.027
	Up to 30	.8096	.0253	
	31-45	.7577	.0376	
	46-149	.7562	.0287	
No. of Other Chronic Conditions (%)	0 (COPD only)	.7754	.0491	F=2.620 p=.039 eta sq.=.086
	1	.7557	.0334	
	2	.8088	.0323	
	3-4	.8002	.0244	
	5+	.6168	.0911	
Medications Tertile (%)	1-5	.8337	.0263	F=6.615 p=.002 eta sq.=.105
	6-8	.7921	.0238	
	9-25	.6832	.0385	
Overall Sample Mean		.7695	.0183	

Table 3: Reported NHS service utilisation over 12-months

Type of healthcare resource use	Number of patients		Mean quantity (SE)*		Mean treatment difference *(Usual care minus SPACE)* (95% CI)
	Usual care	SPACE	Usual care	SPACE	
**NHS SERVICES**					
GP Consultations	59	57	5.00 (0.79)	2.49 (0.32)	2.51(3.42 to 6.58)
Practice Nurse	59	57	1.78 (0.47)	1.44 (0.44)	0.34(0.93 to 1.62)
Psychologist/ Counsellor	59	57	0.58 (0.42)	0.53 (0.37)	0.05(-1.06 to 1.16)
Pulmonary Services	59	57	0.92 (0.39)	0.56 (0.29)	0.35(-0.61 to 1.32)
Inpatient Short Stay	59	57	0.02 (0.02)	0.04 (0.02)	-0.02(-0.08 to 0.04)
Inpatient Long Stay	59	57	0.10 (0.04)	0.11 (0.05)	-0.003(-0.14 to 0.13)
A & E Appointment	59	57	0.59 (0.28)	0.26 (0.08)	0.33(-0.26 to0.92)
**PRESCRIPTIONS**					
Respiratory	59	57	27.65 (2.52)	29.88 (2.46)	-2.22 (-9.21 to 4.76)
Antibiotics	59	57	7.38 (1.37)	8.55 (1.67)	-1.17 (-5.43 to 3.09)
Steroids	59	57	4.52 (0.93)	6.16 (1.44)	-1.64 (-5.02 to 1.74)
All Prescriptions	59	57	99.81 (7.90)	91.46 (5.42)	8.36 (-10.75 to 27.46)

NHS: National Health Service; SPACE: self-management programme of activity, coping and education; SE=standard error; CI=confidence interval; GP= General practitioner

* Missing values replaced using Bayesian Linear Mixed Model, taking into account covariates and confounders

Annual number of prescriptions were calculated by multiplying number of medications prescribed x 13.

Table 4: Unit Costs of Healthcare Resource Use (2021-22)

Item	Unit	Price (£)	Source
GP	*9.22 minute*	35	*Unit Costs of Health and Social Care 2022*
Practice Nurse	*unit cost*	17	*Inflation-adjusted figures (ref 1).*
Psychologist/ Counsellor	*per hour*	66	*Unit Costs of Health and Social Care 2022*
Hospital A & E attendance	*unit cost*	304	*Unit Costs of Health and Social Care 2022*
Hospital Short Stay (1-3)	*unit cost*	845	*Inflation-adjusted figures (ref 1).*
Hospital Long Stay	*unit cost*	4280	*Inflation-adjusted figures (ref 1).*
Pulmonary Service	*unit cost*	372	*NHS Tariff*
Community Exercise Scheme	*unit cost*	302	*Inflation-adjusted figures (ref 2)*
111 helpline,	*unit cost*	13	*Inflation-adjusted figures (ref 3)*
CT scan	*unit cost*	166	*Inflation-adjusted figures (ref 3)*
Hospital admission	*unit cost*	801	*National schedule of NHS costs - Year 2021/22 - NHS*
Oxygen assessment	*unit cost*	293	*National schedule of NHS costs - Year 2021/22 - all NHS - HRG data*
Oxygen nurse at home	*unit cost*	16	*Inflation-adjusted figures (ref 1).*
Pacemaker check	*unit cost*	159	*Inflation-adjusted figures (ref 3)*
Lung Function	*unit cost*	179	*National schedule of NHS costs - Year 2021/22 - all NHS - HRG data*
Pharmacist	*unit cost*	53	*Unit Costs of Health and Social Care 2022*
Pulmonary consultant	*unit cost*	74	*Inflation-adjusted figures (ref 1).*
Respiratory Consultant	*unit cost*	74	*Inflation-adjusted figures (ref 1).*
Respiratory Nurse	*unit cost*	23	*Inflation-adjusted figures (ref 1).*
Respiratory Consultant (phone app)	*unit cost*	74	*Inflation-adjusted figures (ref 1).*
Sleep apnoea appointment	*unit cost*	355	*National Schedule of NHS Costs Year : 2021/22 - All NHS-Outpatient Attendances Data*
Sleep clinic	*unit cost*	355	*National Schedule of NHS Costs Year : 2021/22 - All NHS-Outpatient Attendances Data*
Sleep service,	*unit cost*	355	*National Schedule of NHS Costs Year : 2021/22 - All NHS-Outpatient Attendances Data*

Spirometry	*unit cost*	159	*Inflation-adjusted figures (ref 3)*
Blood test in GP	*unit cost*	7	*Inflation-adjusted figures (ref 3)*
Physiotherapy/Physiotherapist	*unit cost*	92	*Unit Costs of Health and Social Care 2022*
Physiotherapy (MSK)	*unit cost*	92	*Unit Costs of Health and Social Care 2022*
COPD Nurse	*unit cost*	23	*Inflation-adjusted figures (ref 1).*
COPD nurse home visits	*unit cost*	16	*Inflation-adjusted figures (ref 1).*
Community Physiotherapist	*unit cost*	52	*Inflation-adjusted figures (ref 1).*
Dietician	*unit cost*	100	*Unit Costs of Health and Social Care 2022*
Respiratory/sleep studies	*unit cost*	292	*National schedule of NHS costs - Year 2021/22 - all NHS - HRG data*
Hospital appointments	*unit cost*	235	*Unit Costs of Health and Social Care 2022*
Outreach service	*unit cost*	25	*Unit Costs of Health and Social Care 2022*

References:

1. Klonizakis M, Tew GA, Gumber A, Crank H, King B, Middleton G, et al. Supervised exercise training as an adjunct therapy for venous leg ulcers: a randomized controlled feasibility trial. Br J Dermatol. 2018;178(5):1072-82

2. Anokye NK, Trueman P, Green C, Pavey TG, Hillsdon M, Taylor RS. The cost-effectiveness of exercise referral schemes. BMC Public Health. 2011;11:954.

3. Gumber A, Ramaswamy B, Ibbotson R, Ismail M, Thongchundee O, Harrop D, et al. Economic, Social and Financial Cost of Parkinson's on Individuals, Carers and their Families in the UK. Project Report. . Sheffield Hallam University, UK; 2017.
