# Supplementary material for: Cost-effectiveness of a self-management maintenance programme following pulmonary rehabilitation: a UK randomised controlled trial for patients with chronic obstructive pulmonary disease
Source: BMJ Open Respir Res. 2025 Dec 4;12(1):e003406. doi: 10.1136/bmjresp-2025-003406 (PMC12684092; doi:10.1136/bmjresp-2025-003406)

Supplemental Material Appendix 4

Societal Perspective - Cost-Effectiveness

Figure a: Cost-Effectiveness Plane (Societal perspective), bootstrap samples using GLM model

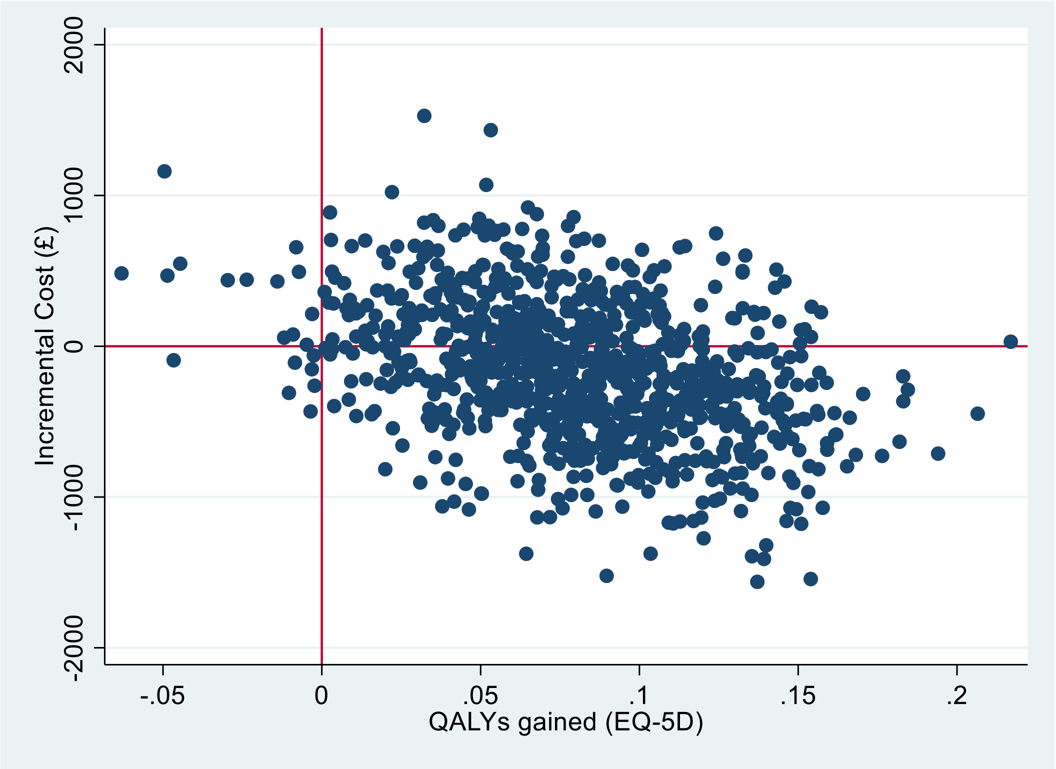

Figure b: Cost-Effectiveness Acceptability Curve (Societal perspective)

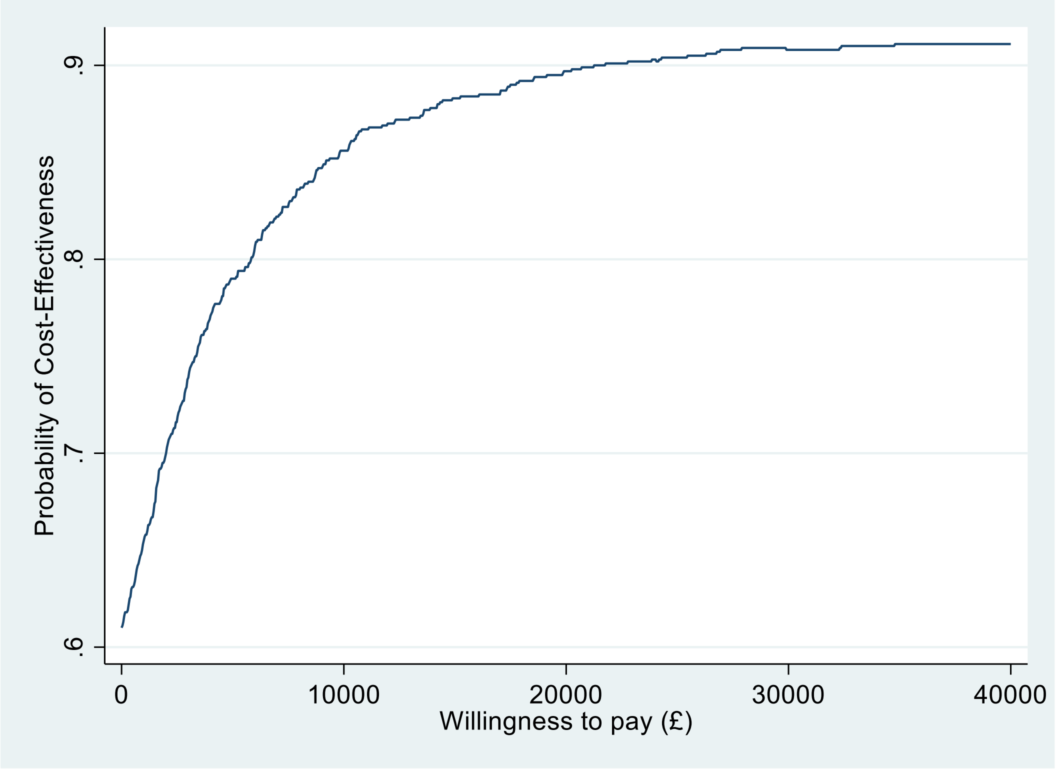

Supplement: online supplemental file 4 [file bmjresp-12-1-s004.pdf]
